# Differential Psychological Factors Associated With Unnecessary Dental Avoidance and Attendance Behavior During the Early COVID-19 Epidemic

**DOI:** 10.3389/fpsyg.2021.555613

**Published:** 2021-05-26

**Authors:** Yi Feng Wen, Peng Fang, Jia-xi Peng, Shengjun Wu, Xufeng Liu, Qian Qian Dong

**Affiliations:** ^1^Key Laboratory of Shaanxi for Craniofacial Precision Medicine Research, College of Stomatology, Xi'an Jiaotong University, Xi'an, China; ^2^Department of Military Medical Psychology, Air Force Medical University, Xi'an, China; ^3^College of Teachers, Chengdu University, Chengdu, China

**Keywords:** dental attendance, dental avoidance, COVID-19 epidemic, psychological characteristics, dental emergency

## Abstract

The coronavirus disease 2019 (COVID-19) pandemic caused by severe acute respiratory syndrome coronavirus 2 is challenging the dental community to an unprecedented degree. Knowledge of the increased risk of infection in dental settings has been disseminated to the public and guidelines have been formulated to assist dental attendance decision-making. However, dental attendance behaviors incompatible with treatment need is not uncommon in clinical settings. Important gaps remain in the knowledge about how psychological factors are affecting dental attendance behaviors during the COVID-19 epidemic. In this cross-sectional study, a questionnaire survey was performed during February and March 2020. A total of 342 and 294 dental patients who attended and avoided dental visits, respectively, were included. The participants were classified into four groups based on dental attendance behavior and emergent/urgent dental treatment need. Bivariate analysis was performed to investigate factors associated with dental attendance. Multivariable logistic regression based on principal component scores was performed to identify major psychological constructs associated with unnecessary dental avoidance and attendance. Among all the factors explored, inability to wear masks during dental treatment (*P* < 0.001; effect size: 0.32) was most closely associated with the overall pattern of dental attendance among participants. Multivariable regression suggested that unnecessary dental avoidance was associated with perceived risk of infection in general and in dental settings (odds ratio [95% CI]: 0.62 [0.53, 0.72]; *p* < 0.001), perceived impact of COVID-19 and dental problems on general health (0.79 [0.65, 0.97]; 0.021), and personal traits such as trust and anxiety (0.77 [0.61, 0.98]; 0.038). Unnecessary dental attendance was associated with optimism toward the epidemic (1.68 [1.42, 2.01]; <0.001) and trust (1.39 [1.13, 1.74]; 0.002). Multidisciplinary efforts involving dental and medical professionals as well as psychologists are warranted to promote more widespread adoption, among the general public, of dental attendance behaviors compatible with dental treatment need during the COVID-19 epidemic.

## Introduction

The spread of severe acute respiratory syndrome coronavirus 2 (SARS-CoV-2), a novel strain of coronavirus of zoonotic origin, since its emergence in early December 2019 has resulted in a global pandemic of coronavirus disease 2019 (COVID-19) (World Health Organization, 2020). This has put the dental community against an unprecedented challenge. Dentists and dental patients in clinical settings are at an increased risk of exposure and infection due to the physical proximity between dentists and patients, generation of large volumes of droplets and aerosol during treatment (Cristina et al., 2008), and the inability of the patients to wear masks during treatment. Moreover, patients can be exposed to cross-contamination in the dental office in the absence of adequate precautions (Izzetti et al., 2020). On the other hand, dental problems such as acute pulpitis, acute apical abscess, and traumatic dental injury can lead to extremely painful symptoms and, if left untreated, may develop into life-threatening conditions. Therefore, patients suffering from dental problems are faced with the dilemma of two choices: either visiting dental care providers to alleviate symptoms at the cost of an increased risk of infection or living with symptoms of dental problems at home.

Government and dental professional associations in China and other countries have issued instructions and guidelines regarding the operation of dental services during the COVID-19 epidemic (Ather et al., 2020). In China, all private dental clinics are required to shut down while only selected public dental hospitals to remain open to sustain dental care services for patients in distinct need of emergency treatment. The Chinese government (Li and Meng, 2020; National Health Committee, 2020) and ADA (American Dental Association) formulated guidelines for patient triage and screening and specified the spectrum of emergent/urgent dental diseases that require immediate treatment [American Dental Association (ADA) (2020)]. Both guidelines were developed with the fundamental principle that only patients with emergent/urgent dental problems should be treated and efforts should be made to minimize the risk of contraction of SARS-CoV-2 in the dental office by dental professionals.

In spite of public advocacy of these government policies and professional guidelines, clinical observation suggested that it was not uncommon for patients without emergent/urgent need of dental care to visit dental hospitals. On the other hand, a large number of patients with distinct needs of immediate care refused to visit dental hospitals. These inadequate dental attendance behaviors impede proper prioritization of dental care resources and unnecessarily increase the risk of SARS-CoV-2 exposure and infection by dental professionals and patients. Dental attendance behaviors are evidently influenced by psychological factors. Dental phobia results in irregular dental care-seeking behavior (Bernson et al., 2013). Likewise, pregnant women tend to be hesitant in terms of dental visits although dental care can lead to improved pregnancy outcomes (Al Habashneh et al., 2005; Wrzosek and Einarson, 2009). Consequently, we hypothesize that inadequate dental attendance behavior during the COVID-19 epidemic is influenced by psychological factors.

At the time of writing, limited studies have discussed the impact of the COVID-19 epidemic on dentistry, and even fewer have examined the impact of the epidemic on the psychological status of dental patients. Zhai and Zhou (2020) reviewed dental diseases associated with psychological status and emphasized the need for dentists to pay attention to the psychological status of dental patients. During the SARS outbreak, over 30% of the dental patients in Hong Kong were worried about being infected from dental treatment (Yip et al., 2007). However, only dental attendees were surveyed in this study. Psychological factors associated with dental avoidance and attendance during major disease epidemics cannot be identified from this study. Therefore, the usefulness of this study was limited in terms of informing individualized, targeted psychological interventions that promote the adoption of adequate dental attendance behavior.

In addition to perception toward COVID-19 and perception toward dental visits during this period, coping strategy, anxiety, and trust were also potentially associated with dental attendance behavior. The coping strategy changes the assessment of stress events and regulates physical and emotional responses related to the event (Li et al., 2014). The relationship between individual coping style and mental and physical health has become an important research area in clinical psychology. Previous studies have shown that coping style was related to mental health (Yu et al., 2007). Social anxiety may lead to hesitant behavior, avoidance, and performance difficulties and is therefore potentially associated with reluctant dental-seeking behavior (Maner et al., 2007). In addition, we investigated the trust of the respondents toward others and toward health care workers separately since they may differentially impact dental attendance behavior (Trachtenberg et al., 2005).

The present study aimed at comprehensively assessing psychological factors belonging to multiple domains that influence the attendance behavior of dental patients during the COVID-19 epidemic. Emphasis was placed on psychological factors that are, respectively, associated with unnecessary dental avoidance and attendance. Identification of such psychological determinants would help address inadequate dental attendance behavior and promote widespread adoption of dental attendance behavior compatible with real dental treatment needs of the patients during the COVID-19 epidemic.

## Methods

### Research Approach

A cross-sectional questionnaire survey was performed to investigate psychological factors associated with dental attendance behavior during the early stage of the COVID-19 epidemic. Analyses were performed separately for each type of dental attendance behavior to investigate how dental attendance behavior is associated with varying psychological factors.

### Procedure and Participants

Participants consisted of 636 adults age 18–80 years who had varying severity of dental diseases during February and March 2020, when the COVID-19 epidemic was at its peak in China. A total of 355 participants who visited the Hospital of Stomatology of Xi'an Jiaotong University, a teaching hospital that remained functional throughout the epidemic period, were recruited. To recruit potential dental patients who did not visit the dental hospital, questionnaires were distributed through WeChat, the largest social media platform in China, covering populations of varying ages, and a total of 946 individuals responded. Among the respondents, 281 were identified as having dental problems and were therefore included in the analysis conducted. Patients were excluded if they themselves or their dependents were unable to cooperate to complete the questionnaire online. Ethics approval was obtained from the Clinical Research Ethics Committee of the Hospital of Stomatology of Xi'an Jiaotong University. Participants gave informed consent prior to their participation.

On the basis of Chinese government policies and guidance from ADA, which are essentially in mutual agreement, we classified participants into four groups according to their emergent/urgent treatment needs and dental attendance behavior (Table 1): patients with emergent/urgent dental treatment needs who visited dentists (T-V); patients with emergent/urgent dental treatment needs who did not visit dentists (T-nV); patients without emergent/urgent dental treatment needs who visited dentists (nT-V); and patients without emergent/urgent dental treatment needs who did not visit dentists (nT-nV). Participants in groups T-V and nT-nV demonstrated dental attendance behaviors compatible with their treatment needs and were combined into a single group, termed group C (C for compatible), during further analysis.

**Table 1:** Criteria for classification of participants.

	**T-V**	**T-nV**	**nT-V**	**nT-nV**
Level of pain ≥7/trauma/localized swelling	+	+	–	–
Dental visit	+	–	+	–
Hypothetical question	+	NA	NA	+

*For the hypothetical question, participants in group T-V were asked if they would still visit dentists if their symptoms do not constitute dental emergency/urgency. Participants in group nT-nV were asked if they would still avoid visiting dentists if their symptoms constitute dental emergency/urgency*.

### Measures

**Figure 1:**
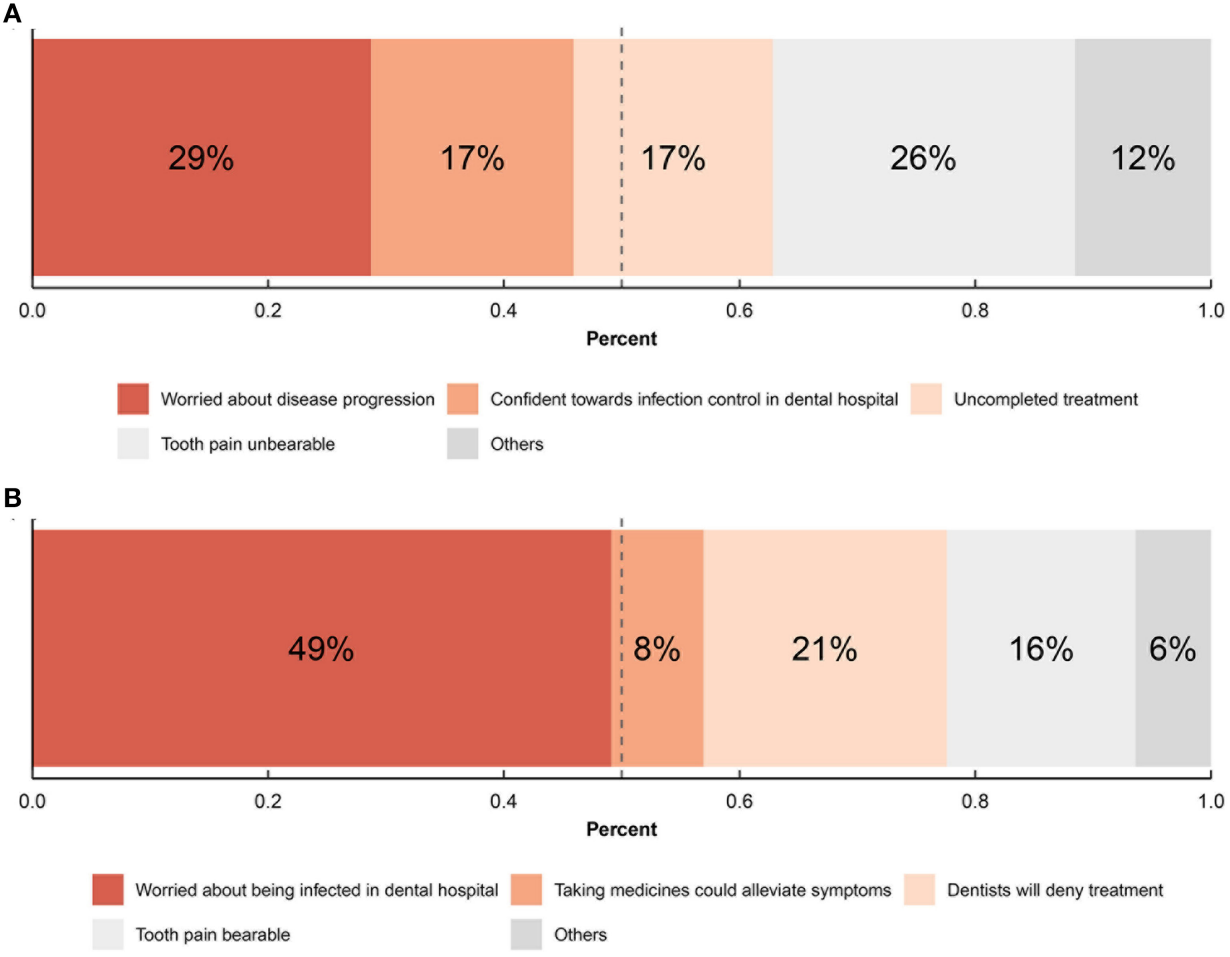
Distributions of reasons for dental attendance and avoidance. **(A)** Distribution of reasons of dental attendance among respondents who visited dentists; **(B)** distribution of reasons of dental avoidance among respondents who did not visit dentists.

All participants responded to the questionnaire (Supplementary Table 1) online. The demographic and socioeconomic characteristics of the participants were recorded at the beginning of the questionnaire (Q1–4). The occupation of the participants was classified into occupational groups, namely I (professional), II (managerial and technical), III (skilled), IV (partly skilled), and V (unskilled), according to the Registrar-General's Social Classes (Bland, 1979). Reasons for dental attendance and avoidance were also included (Q6–10). Distribution of participants by reasons of attendance/avoidance provides an understanding of the impact of psychology-related factors on the dental attendance behavior (Figure 1).

The remainder of the questionnaire involved five major domains. The first domain was designed to evaluate the cognition, emotion, and behavior of the participants (Q14–22) to the epidemic. Cognition and emotion significantly affect human behavior (Dolan, 2002). Cognition and emotion-related questions were incorporated into the questionnaire based on questions from a large-scale study on the perception of the general public toward COVID-19 among Chinese citizens (Fang et al., 2020). Cognitive questions involved views on the current status and future trends of the epidemic and knowledge about dental diseases. Emotional questions mainly captured emotional reactions. Behavior-related questions evaluated the perceived impact of the epidemic on daily lives. The second domain investigated knowledge and attitude toward dental attendance during the COVID-19 epidemic based on questions developed by dental experts from different fields (Q23–28). Trust in People Questionnaire (TPQ), which assessed how individuals trust others (Robinson et al., 1973), was included as the third domain of the questionnaire (Q29–35). We additionally included a question (Q32) on the trust of the respondents toward health care providers (Trachtenberg et al., 2005). The Simplified Coping Style Questionnaire (SCSQ) in the fourth domain (Q36–55) of the questionnaire, which assessed individuals' consciousness, purposefulness, and flexibility in regulating behaviors in the real environment, was developed by Xie (1998) on the basis of the Ways of Coping questionnaire by Folkman and Lazarus (1988). SCSQ is a 20-item self-administered questionnaire, including 12 positive response items and 8 negative response items. A higher score represents a more positive/negative coping style. Subjects were asked to indicate their level of agreement on the four-point Likert scale based on the frequency of their adoption of items ranging from 0 (“Never”) to 3 (“Very frequent”). The questionnaire has been proven to be reliable and effective and widely used in China (Li et al., 2014). The fifth domain (Q56–62), the Interaction Anxiousness Scale (IAS), assessed the tendency of the subjective social anxiety experience independent of behavior. IAS is a self-reported measure of dispositional social anxiety with proven reliability and validity (Leary and Kowalski, 1993). The IAS contains 15 items, which are answered on a 5-level scale (1 = Does not match me at all; 2 = Matches me a little bit; 3 = Agrees with me to a moderate degree; 4 = Consistent with me; 5 = Very consistent with me). Its total score ranges from 15 (the lowest level of social anxiety) to 75 (the highest level of social anxiety). Cronbach's alpha was calculated for each of the five domains using the “alpha” function from the R package “psych.” The values of Cronbach's alpha were 0.61, 0.51, 0.62, 0.85, and 0.79 from the first to the fifth domain, respectively.

A question on the degree of anxiety toward dental visits before the epidemic was used to identify and exclude participants with dental phobia (Q13). A question was designed to exclude all respondents without any dental problems (Q5). It is noteworthy that participants in the T-V group may still visit dentists if their dental conditions were not as emergent/urgent, and *vice versa* for participants in the nT-nV group. To ensure that the T-V and nT-nV groups included only participants whose perceived need toward dental visit is compatible with the emergency/urgency of the dental condition, two hypothetical questions were asked. In these two questions, participants belonging to the T-V group were asked to indicate if they would still visit dentists if their dental conditions were not emergent/urgent (Q11). A similar question was asked to participants belonging to the nT-nV group (Q12).

### Statistical Analysis

The distribution of participants by demographic and socioeconomic characteristics was described for each behavioral group. The distribution of reasons for dental attendance and avoidance were evaluated. ANOVA was performed to investigate mean differences in response to each question among the four dental attendance behavior groups. The effect size of ANOVA was determined by $\eta =\sqrt{\frac{SS_{M}}{SS_{T}}}$, where *SS*_*M*_ is the model sum of square and *SS*_*T*_ is the total sum of square. Furthermore, response to each question was compared between the T-nV and C groups, as well as between the nT-V and C groups, through planned contrasts. In the first contrast, group T-nV is assigned a weight of 2, nT-V is assigned a weight of 0, T-V and nT-nV are each assigned a weight of −1. This allows for the comparison of groups T-nV and C. In the second contrast, group T-nV is assigned a weight of 0, nT-V is assigned a weight of 2, and T-V and nT-nV are each assigned a weight of −1. This allows for the comparison of groups nT-V and C. Effect size of planned contrast was determined by $r=\sqrt{\frac{t^{2}}{t^{2}+df}}$ (Rosenthal, 1994). Following Cohen's guidelines, 0.3 and 0.5 were used as the threshold for small, medium, and large effect for both η and *r*, respectively. Categorical variables were compared among and between groups through a chi-square test with effect size estimated through Cramér's V.

Factors significantly associated with dental attendance behaviors in bivariate analysis (ANOVA and planned contrast) were then jointly examined through multivariable logistic regression. Logistic regression models were established separately for T-nV vs. C and nT-V vs. C. Since items in the questionnaires are not mutually independent, principal component analysis (PCA) was performed to extract principal components (PCs) from those significant items. The “prcomp” function in R was used for the PCA and the calculation of PC scores. Multivariable logistic regression was then performed based on individual PC scores. All tests were two-sided and the level of statistical significance was set at 0.05. ANOVA and logistic regressions were performed using R software (version 4.0.2). The “contrasts” function in R was used for the construction of contrasts and the “aov” function was used for ANOVA. Logistic regressions were performed using the “glm” function with the logit link. All functions used in this study are available in base R.

## Results

### Demographic and Socioeconomic Characteristics

Completed questionnaires were received from 636 surveyed participants. Among the participants, 44% were males. Young people (age 18–30) accounted for 35%, middle-aged people (age 30–59) accounted for 24%, and the elderly (age 60–80) accounted for 10%. There were 63% of respondents who had an undergraduate degree or above. Skilled workers (Occupation group III) had the largest proportion of participants (35.7%) among all occupational categories. The distributions of each characteristic among the four behavioral groups are described in Table 2. It was found that 63 and 78% of dental avoidance and attendance behaviors of the participants, respectively, were psychologically related.

**Table 2:** Demographic and socioeconomic characteristics of respondents.

	**T-V**	**T-nV**	**nT-V**	**nT-nV**
**Sex**				
Female	103 (52.28%)	82 (54.3%)	93 (58.86%)	76 (58.46%)
Male	94 (47.72%)	69 (45.7%)	65 (41.14%)	54 (41.54%)
**Age**				
18–29 years	72 (36.55%)	47 (31.13%)	51 (32.28%)	52 (40%)
30–59 years	101 (51.27%)	90 (59.6%)	98 (62.03%)	63 (48.46%)
60–80 years	24 (12.18%)	14 (9.27%)	9 (5.7%)	15 (11.54%)
**Education level**				
Secondary school or lower	33 (16.75%)	35 (23.18%)	26 (16.46%)	22 (16.92%)
Undergraduate	140 (71.07%)	85 (56.29%)	99 (62.66%)	78 (60%)
Postgraduate	24 (12.18%)	31 (20.53%)	33 (20.89%)	30 (23.08%)
**Occupation**				
I	28 (14.21%)	26 (17.22%)	33 (20.89%)	28 (21.54%)
II	25 (12.69%)	29 (19.21%)	27 (17.09%)	22 (16.92%)
III	77 (39.09%)	48 (31.79%)	58 (36.71%)	44 (33.85%)
IV	27 (13.71%)	23 (15.23%)	16 (10.13%)	12 (9.23%)
V	40 (20.3%)	25 (16.56%)	24 (15.19%)	24 (18.46%)
**Friends being confirmed, suspected, or isolated**				
Confirmed	0 (0%)	4 (2.65%)	0 (0%)	0 (0%)
Suspected	3 (1.52%)	2 (1.32%)	1 (0.63%)	0 (0%)
Isolated	3 (1.52%)	3 (1.99%)	0 (0%)	9 (6.92%)
None of the above	191 (96.95%)	142 (94.04%)	157 (99.37%)	121 (93.08%)
**Participant being confirmed, suspected, or isolated**				
Confirmed	0 (0%)	1 (0.66%)	0 (0%)	0 (0%)
Suspected	0 (0%)	3 (1.99%)	0 (0%)	1 (0.77%)
Isolated	0 (0%)	4 (2.65%)	2 (1.27%)	3 (2.31%)
None of the above	197 (100%)	143 (94.7%)	156 (98.73%)	126 (96.92%)

### Factors Associated With the Pattern of Dental Attendance During the COVID-19 Epidemic

**Figure 2:**
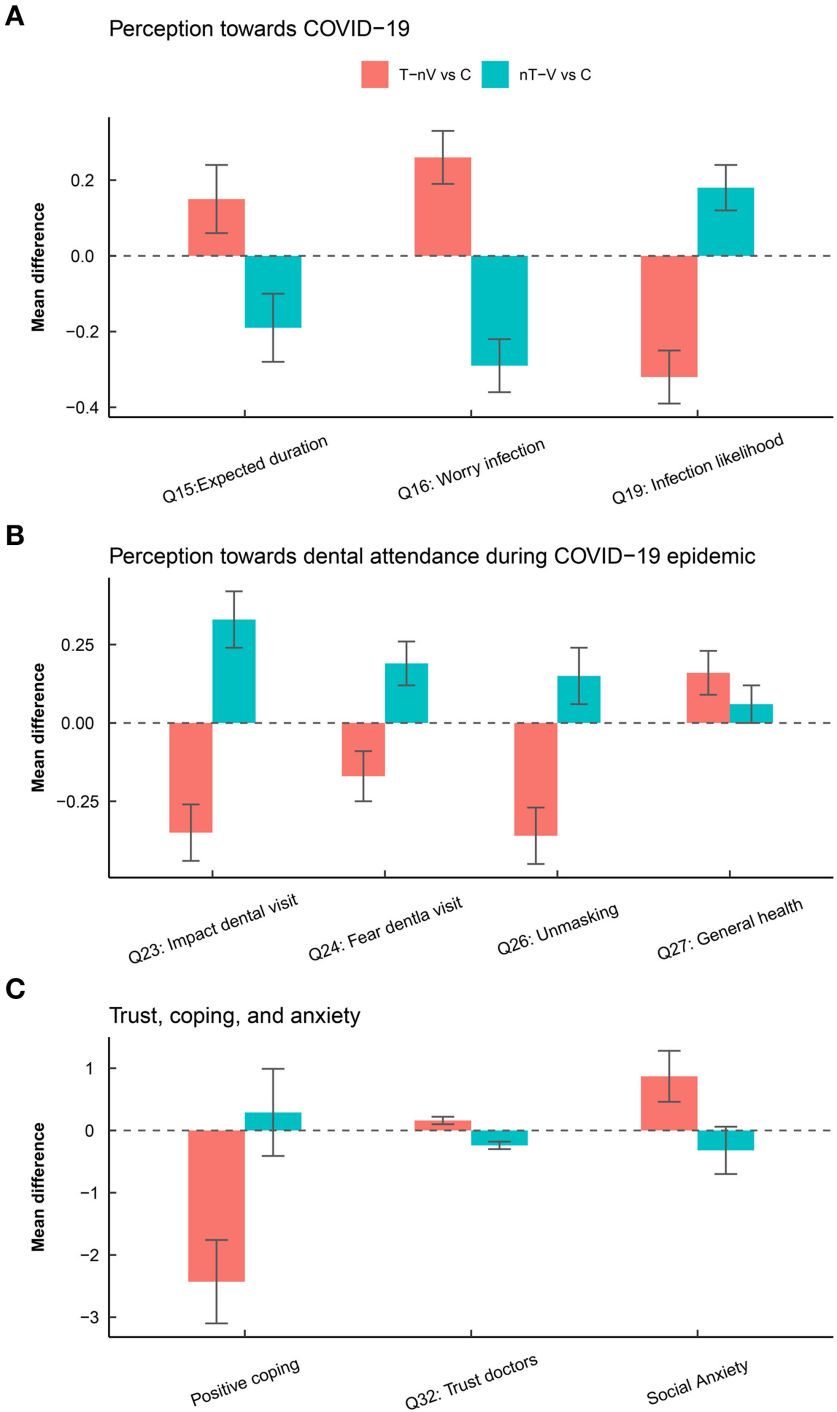
Differences in the response of participants among the four behavioral groups. **(A)** Difference in perception toward COVID-19. **(B)** Differences in perception toward dental attendance during the COVID-19 epidemic. **(C)** Differences in coping, trust, and social anxiety.

The ANOVA and planned contrast identified factors associated with dental attendance behavior from each of the five domains in the questionnaire (Table 3, Figure 2). The behavior of being worrisome about getting infected was most closely associated with the overall pattern of dental attendance (*P* < 0.001; ES = 0.28) and was significantly associated with both unnecessary dental avoidance (<0.001; 0.16) and attendance (<0.001; 0.19). Perceived likelihood of being infected was likewise associated with both unnecessary dental avoidance (<0.001; 0.20) and attendance (<0.001; 0.12) behaviors. The expected epidemic duration was uniquely associated with unnecessary dental attendance behavior (0.01; 0.10). Considering dental hospital to be the most dangerous place was uniquely associated with dental avoidance behavior (<0.001; 0.12). Perceived impact of the epidemic on dental visits (<0.001; 0.31) and degree of fearfulness toward dental visits (<0.001; 0.16) were associated with the general pattern of dental attendance. Unmasking during dental visits was most closely associated with the pattern of dental attendance (<0.001; 0.32) but further analysis revealed that it was only associated with unnecessary dental avoidance (<0.001; 0.18). The perceived impact of oral health on general health was significantly associated with only unnecessary dental avoidance (0.01; 0.10). Trust toward health care providers was associated with both unnecessary dental avoidance (<0.001; 0.11) and attendance (<0.001; 0.18). Positive coping, on the other hand, was uniquely associated with unnecessary dental avoidance (<0.001; 0.16). In terms of anxiety, trait anxiety (0.002; 0.13) and social anxiety (0.01; 0.10) were uniquely associated with unnecessary dental avoidance. Table 3 suggests that participants who avoided necessary dental visits differed from group C in the reverse direction in which participants who unnecessarily visited dental hospitals differed from group C.

**Table 3:** Factors associated with dental attendance during the COVID-19 epidemic.

	**Mean difference (SE)**	***P*-value**	**ES**
**DOMAIN A: PERCEPTION TOWARD COVID-19**			
**Q15: Expected duration of the epidemic**			
Overall		0.002^**^	0.15
T-nV vs. C	0.15 (0.09)	0.080	0.07
nT-V vs. C	−0.19 (0.09)	0.01^*^	0.10
**Q16: Worried about being infected**			
Overall		<0.001^***^	0.28
T-nV vs. C	0.26 (0.07)	<0.001^***^	0.16
nT-V vs. C	−0.29 (0.07)	<0.001^***^	0.19
**Q19: Self-perceived likelihood of being infected**			
Overall		<0.001^***^	0.27
T-nV vs. C	−0.32 (0.07)	<0.001^***^	0.20
nT-V vs. C	0.18 (0.06)	<0.001^***^	0.12
**Q22: Most dangerous place**			
Overall		<0.001^***^	0.08
T-nV vs. C		<0.001^***^	0.12
nT-V vs. C		0.12	0.05
**DOMAIN B: PERCEPTION TOWARD DENTAL ATTENDANCE DURING COVID-19 EPIDEMIC**			
**Q23: Impact on dental visit**			
Overall		<0.001^***^	0.31
T-nV vs. C	−0.35 (0.09)	<0.001^***^	0.18
nT-V vs. C	0.33 (0.09)	<0.001^***^	0.17
**Q24: Degree of fearfulness toward dental visit**			
Overall		<0.001^***^	0.16
T-nV vs. C	−0.17 (0.08)	0.017^*^	0.10
nT-V vs. C	0.19 (0.07)	<0.001^***^	0.12
**Q26: Unmasking during dental treatment increases likelihood of being infected**			
Overall		<0.001^***^	0.32
T-nV vs. C	−0.36 (0.09)	<0.001^***^	0.18
nT-V vs. C	0.15 (0.09)	0.25	0.08
**Q27: Oral health impacts general health**			
Overall		0.08	0.10
T-nV vs. C	0.16 (0.07)	0.01^*^	0.10
nT-V vs. C	0.06 (0.06)	0.31	0.05
**DOMAIN C: TRUST**			
**Q32: Trust toward health care providers**			
Overall		<0.001^***^	0.23
T-nV vs. C	0.16 (0.06)	<0.001^***^	0.11
nT-V vs. C	−0.24 (0.06)	<0.001^***^	0.18
**DOMAIN D: COPING**			
**Positive coping inventory**			
Overall		0.001^**^	0.16
T-nV vs. C	−2.43 (0.67)	<0.001^***^	0.16
nT-V vs. C	0.29 (0.70)	0.68	0.02
**DOMAIN E: ANXIETY**			
**Q56: Trait anxiety**			
Overall		<0.001^***^	0.12
T-nV vs. C		0.002^**^	0.13
nT-V vs. C		0.14	0.06
**Social anxiety inventory**			
Overall		0.008^**^	0.14
T-nV vs. C	0.87 (0.41)	0.01^*^	0.10
nT-V vs. C	−0.32 (0.38)	0.24	0.04

*Mean differences were presented for all questions except for questions with categorical responses. SE, standard error; ES, effect size. When groups T-nV and nT-V are compared against group C, mean values for group C were subtracted from those for groups T-nV and nT-V, respectively*.

* *p < 0.05*;

** *p < 0.01*;

*** *p < 0.001*.

Multivariable logistic regression based on group T-nV and group C (Table 4, Supplementary Table 2) suggested that higher PC1 scores, which represented a lower perceived risk of SARS-CoV-2 infection in dental hospitals, was associated with reduced likelihood of unnecessary dental avoidance (odds ratio [95% CI]: 0.62 [0.53, 0.72]; *p* < 0.001). Participants with higher scores along PC3, which was indicative of the less perceived impact of COVID-19 and greater impact of dental problems toward general health, were less likely to avoid necessary dental visits (0.79 [0.65, 0.97]; 0.021). PC6, which was associated with positive personal traits such as the adoption of positive coping and less social anxiety, was associated with reduced likelihood of unnecessary dental avoidance (0.77 [0.61, 0.98]; 0.038). Multivariable logistic regression based on group nT-V and group C (Table 5, Supplementary Table 3) suggested that higher PC1 scores, indicative of a positive perception toward the COVID-19 epidemic, increased the likelihood of unnecessary dental attendance (1.68 [1.42, 2.01]; <0.001). PC2, which accounted for increased trust toward health care providers and less fearfulness toward dental visits, increased the likelihood of unnecessary dental visits by 39% (1.39 [1.13, 1.74]; 0.002).

**Table 4:** Multivariable associations between principal components of psychological factors and dental avoidance.

	**OR (95% CI)**	***P*-value**
PC1 (Perceived increased risk of SARS-CoV-2 infection in dental hospital)	0.62 (0.53, 0.72)	<0.001^***^
PC2	1.13 (0.95, 1.34)	0.175
PC3 (Perceived relative importance of COVID-19 vs. dental problems on general health)	0.79 (0.65, 0.97)	0.021^*^
PC4	0.87 (0.71, 1.07)	0.188
PC5	0.90 (0.73, 1.12)	0.366
PC6 (Personal trait)	0.77 (0.61, 0.98)	0.038^*^
PC7	1.06 (0.84, 1.35)	0.626
PC8	0.91 (0.70, 1.19)	0.491
PC9	1.05 (0.79, 1.40)	0.718

*OR, odds ratio; 95% CI, 95% confidence interval. Participants in group C were coded as 0 and in group T-nV were coded as 1*.

* *p < 0.05*;

*** *p < 0.001*.

**Table 5:** Multivariable associations between principal components of psychological factors and unnecessary dental attendance.

	**OR (95% CI)**	***P*-value**
PC1 (Judgement of COVID-19 epidemic)	1.68 (1.42, 2.01)	<0.001^***^
PC2 (Trust toward others)	1.39 (1.13, 1.74)	0.002^**^
PC3	0.89 (0.71, 1.11)	0.310
PC4	0.98 (0.79, 1.21)	0.840
PC5	0.99 (0.78, 1.26)	0.914
PC6	1.03 (0.80, 1.34)	0.813

*OR, odds ratio; 95% CI, 95% confidence interval. Participants in group C were coded as 0 and in group nT-V were coded as 1*.

** *p < 0.01*;

*** *p < 0.001.*.

## Discussion

To the knowledge of the authors, this is the first study that has comprehensively investigated the impact of psychological factors on the patterns of dental attendance behavior during the COVID-19 epidemic. By separately analyzing psychological factors influencing unnecessary dental avoidance and attendance, we were able to provide evidence on psychological factors that differentially influence these two dental attendance behaviors.

This study was performed in the city of Xi'an, a prominent city in northwest China whose epidemiological profile of COVID-19 ranked middle among all major cities in China. The study was performed during February and March when the COVID-19 epidemic was at its height in China. Distributions of demographic and socioeconomic characteristics were comparable across the four groups of participants. The findings presented herein are therefore generalizable to the local population. However, care should be taken when extrapolating the present findings to the wider population because differences in the degree of severity of COVID-19 may interact with psychological determinants to affect dental attendance behavior. Whether and how cultural and socioeconomic differences across regions and nations impact dental attendance behavior in addition to psychological factors is worthy of further investigation.

Dental care–seeking behavior is most commonly influenced by dental symptoms. However, the impact of psychological factors on dental attendance should not be neglected. The presented findings suggest that the dental avoidance and attendance behavior of more than half of the participants during the COVID-19 epidemic was psychologically related. This highlighted the importance of psychological factors on dental visits during the COVID-19 epidemic.

Further analyses revealed that participants who unnecessarily avoided dental visits responded to these questions in the reverse direction in which those who unnecessarily attended dental visits responded. This lends further support to the regulatory role of psychological factors in driving dental attendance behaviors. Such findings also justified the need to analyze the groups T-nV and nT-V separately. However, a close examination revealed that factors leading to unnecessary dental avoidance and attendance were not exactly the same. This points toward the complex pathways in which multiple psychological factors interact and ultimately affect dental attendance behaviors.

Multivariable logistic regression identified three components that were associated with unnecessary dental avoidance behavior. The first component characterized individual perception toward the risk of infection both in general and inside dental hospitals. Knowledge about the routes and dynamics of SARS-CoV-2 transmission was limited during the early stage of the COVID-19 epidemic, which resulted in prevalent anxiety and a feeling of uncertainty among the general public. It was estimated that around 70% of the public were worried or terrified of the epidemic. This creates the possibility for cognitive bias to prevail among the general public (Fang et al., 2020). Chinese health authorities have advised the public to use masks since the early stage of the epidemic. Compared to individuals in other countries, especially westerners, masks are given greater importance in their role in individual protection during the COVID-19 epidemic by Chinese individuals. This likely explains the contribution of worry toward unmasking during dental treatment to the first component. As a result, perception toward the epidemic and toward dental visits jointly influenced unnecessary dental avoidance behavior. Dissemination of the knowledge of COVID-19 and timely updates of epidemic status could help relieve the feeling of uncertainty and terror by the general public. Dental patients should be educated that necessary dental treatment should nevertheless be delivered as long as necessary precautions are taken. The strict measures of infection control taken by the dental hospitals should be made aware to the public. Education of this kind could serve as a strategy during cognitive behavioral therapy to help those who would have avoided dental visits to assume a more evidence-based stance toward COVID-19 that is less affected by cognitive bias.

The perceived risk of COVID-19 and dental problems toward general health also impacted dental avoidance. Individuals avoiding necessary dental visits tended to overestimate the impact of COVID-19 and underestimate the impact of dental problems on general health. Expert opinions converge to indicate an uncertain to low risk of SARS-CoV-2 infection in dental hospitals under the condition of adequate precautionary measures taken by both dental hospitals and dental patients. In contrast, there exists a real and distinct risk for dental emergencies to progress into a general health threat. Therefore, to correct for the misjudgment of patients, coordinated efforts from medical and dental health care workers are required to disseminate evidence-based knowledge of the impact of both COVID-19 and dental problems on general health; the emphasis should be placed on the impact of both COVID-19 and dental problems on general health during the consultation and psychological intervention. Greater awareness of general health impact from both aspects would enable the general public to make better-informed dental visit decisions and reduce unnecessary dental avoidance.

Another component associated with dental avoidance involved coping and anxiety. Coping styles directly impact the emotional and physical outcomes of stressful events (Beutler et al., 2011). Positive coping styles affect emotional regulation and facilitate behavioral regulation (Iwata, 2002; MacNeil et al., 2012). A meta-analysis suggests that coping style plays an important role in deciding whether to accept medical and psychological therapeutic interventions (Glanz et al., 2008; Beutler et al., 2011). During the SARS epidemic, positive coping was found to be associated with increased willingness to accept medical treatment and decreased incidence of mental illness (Sim et al., 2010). The findings by the authors likewise identified an association between dental attendance behavior and positive coping, which expands the current knowledge on the impact of coping styles on treatment-related decision-making. Furthermore, the observed findings suggest that strategies that boost positive coping, such as meditation and exercise, may play an essential role in alleviating stress and reducing trepidation. Social anxiety, on the other hand, may lead to hesitant behavior, avoidance, and performance difficulties (Oakman et al., 2003). Increased social anxiety is therefore associated with more frequent unnecessary dental avoidance.

Multivariable logistic regression identified two components associated with unnecessary dental attendance. The first component reflected optimism/pessimism toward the COVID-19 epidemic. Participants unnecessarily attending dental visits were more likely to be optimistic toward the epidemic. Although such optimism is not to be discouraged, proper education is necessary for this population so that limited dental care resources are more effectively delivered to those truly in need. In addition, the presence of this group of population in dental hospitals highlights the importance of the unrelenting effort in infection control in dental hospitals to prevent nosocomial spread of infection because these overly optimistic patients are more likely to be ignorant regarding personal protection. Another component associated with unnecessary dental attendance was trust toward health care providers and the resultant decrease in fear of dental attendance. Like optimism, trust toward health care provides is in no way to be discouraged; better education of the risks of unnecessary dental attendance during the COVID-19 epidemic is critical to this population.

The observed findings suggest that cognitive regulation and knowledge dissemination would help the general public adopt dental attendance behaviors compatible with their real treatment needs. In addition, the promotion of the infection control measures taken by dental hospitals could help offset the perceived risk of unmasking during dental treatment to reduce unnecessary dental avoidance. Methods to boost coping behaviors are also likely to reduce unnecessary dental avoidance. Education about the risk of dental attendance is important in restraining those who planned unnecessary dental visits at home. All of the above strategies require efforts by dental professionals, as well as medical and psychological experts, to actively engage in the decisions of patients toward dental visits. Websites and hotlines maintained by professionals may provide a means to identify the need of each individual so as to develop individualized opinions and, when necessary, psychological interventional strategies, to better serve the general public.

Several limitations of the study bear noting. First, trait anxiety and the perception of the dental hospital being the most dangerous place in terms of being infected were not included in multivariable logistic regression although they were found to be significantly associated with patterns of dental attendance. This is because the answers to these two questions are categorial instead of ordinal or continuous. There is no way in which these questions could be included in PCA. However, we have included other questions of the same domain as these two questions, and hence, the results are not likely to be significantly biased. Second, the findings may be subject to the reporting bias of participants. Classification of dental patients who avoided dental visits with regard to emergent/urgent treatment need was performed purely based on self-reported signs and symptoms. This constitutes a potential source of bias since the rating of the severity of pain may be subject to subjectivity and recall bias. Besides, since the participants of this study were suffering from varying severities of dental symptoms, their response to the questionnaire may be biased by an emotional response to dental symptoms. We included two hypothetical questions at the outset that required participants to indicate their likely dental attendance decision if the severity of their symptoms is different. These hypothetical questions required the devotion and thinking of the participants, which could help the participants calm down and respond to the rest of the questionnaire more sensibly. Third, factors that may influence the strength of the impact of psychological factors on dental attendance behaviors, such as regional level of risk, medical history, and the number of visits, were not taken into consideration. Inclusion of these factors in future studies is likely to produce more robust findings unaffected by variations in these variables. Fourth, the domain of “Perception toward dental attendance during COVID-19 epidemic” is composed of questions that have not been previously validated. These questions were prepared by the joint effort of a team of dental specialists and psychologists with experience in questionnaire development. However, Cronbach's alpha suggested a lower level of internal consistency for this domain relative to other domains. The level of internal consistency is not unacceptable (Cronbach's alpha of <0.5 is considered unacceptable) but we do acknowledge that better-structured questionnaires would provide more valid and robust findings. Given the timeliness of this study, the presented findings are still useful in identifying psychological factors associated with dental attendance during the COVID-19 epidemic.

## Conclusion

Psychological factors play an important role in influencing dental attendance behavior during the COVID-19 epidemic. The perceived risk of SARS-CoV-2 infection in general and in dental hospitals, the perceived impact of COVID-19 and dental problems on general health, and personal traits such as coping style and anxiety influence unnecessary dental avoidance. Unnecessary dental attendance, on the other hand, is driven by optimism/pessimism toward the COVID-19 epidemic and trust toward health care providers. Multidisciplinary efforts are required to better educate and serve the general public and to promote more widespread adoption of dental attendance behavior compatible with dental treatment needs during the current COVID-19 epidemic.

## Data Availability Statement

The raw data supporting the conclusions of this article will be made available by the authors, without undue reservation.

## Ethics Statement

The studies involving human participants were reviewed and approved by the Clinical Research Ethics Committee of Hospital of Stomatology of Xi'an Jiaotong University. The patients/participants provided their written informed consent to participate in this study.

## Author Contributions

YW collected data, performed the statistical analysis, and drafted the manuscript. PF collected data, performed the statistical analysis, and revised the manuscript. J-xP, SW, and XL designed the questionnaire, participated in statistical analysis, and critically reviewed the manuscript. QD conceived the study, designed the study, supervised the data collection process, advised on statistical analysis, and critically revised the manuscript. All authors agree to be accountable for the content of the work.

## Conflict of Interest

The authors declare that the research was conducted in the absence of any commercial or financial relationships that could be construed as a potential conflict of interest. The reviewer MZ declared a shared affiliation with several of the authors, PF, SW, and XL, to the handling editor at time of review.
