# Supplementary material for: Differential Psychological Factors Associated With Unnecessary Dental Avoidance and Attendance Behavior During the Early COVID-19 Epidemic
Source: Front Psychol. 2021 May 26;12:555613. doi: 10.3389/fpsyg.2021.555613 (PMC8187600; doi:10.3389/fpsyg.2021.555613)
Supplement: Supplementary file 1 [file Table_1.DOCX]

Supplementary Material

**Supplementary Table 1. Questionnaire administered by the participants**

| Q1. Gender | |
| --- | --- |
|  | A. Males |
|  | B. Females |
| Q2. You are aged ___ years | |
| Q3. Your occupation belongs to which of the following? | |
|  | A. Student |
|  | B. Government officer |
|  | C. Company manager |
|  | D. Clerk |
|  | E. Professional worker |
|  | F. Worker |
|  | G. Salesman/saleswoman |
|  | H. Self-employed |
|  | I. Agricultural worker |
|  | J. Retired |
|  | K. Unemployed |
|  | L. Others |
| Q4. What is your level of education? | |
|  | A. Junior high school and below |
|  | B. Senior high school |
|  | C. College diploma/undergraduate degree |
|  | D. Postgraduate |
| Q5. Have you experienced tooth pain recently? | |
|  | A. Yes |
|  | B. No |
| Q6. Please indicate your level of pain | |
|  | A. Mosquito bite-like |
|  | B. Very slight pain |
|  | C. Slight pain |
|  | D. Slight to mild pain |
|  | E. Mild pain |
|  | F. Moderate pain |
|  | G. Pain affecting sleep at night |
|  | H. Severe pain |
|  | I. Very severe pain |
|  | J. Worst imaginable pain |
| Q7. Have you experienced facial or intra-oral swelling or dental trauma? | |
|  | A. Yes |
|  | B. No |
| Q8. Have you visited dental hospital? | |
|  | A. Already visited or planning to visit dental hospital |
|  | B. Dare not visit dental hospital |
|  | C. No need to visit dental hospital |
| Q9. What is the major reason of your dental visit | |
|  | A. Unbearable tooth pain |
|  | B. Tooth pain bearable but afraid of disease progression |
|  | C. Tooth pain bearable and trust that dental hospital has taken adequate precautions |
|  | D. Previous treatment uncompleted |
|  | E. Others |
| Q10. What is the major reason of your dental avoidance? | |
|  | A. Tooth pain bearable |
|  | B. Worried about being infected in dental hospital |
|  | C. Taking medicines could alleviate painful symptoms |
|  | D. Dentists will deny treatment during COVID-19 epidemic |
|  | E. Others |
| Q11. Will you still visit dentists if your pain level is below 7 and are free from conditions described in Q7? | |
|  | A. Yes |
|  | B. No |
| Q12. Will you still avoid dental visit if your pain level is 7 or above or have conditions described in Q7? | |
|  | A. Yes |
|  | B. No |
| Q13. Were you anxious about visiting dentists before current COVID-19 epidemic? | |
|  | A. Very anxious. I do not visit dentists even when I have severe tooth pain |
|  | B. Somewhat anxious, but will still visit dentists if I have toothache |
|  | C. Not anxious at all |
| Q14. What is your preceived relevance to COVID-19 epidemic? | |
|  | A. Very relevant |
|  | B. Relevant |
|  | C. Irrelevant |
| Q15. When do you expect COVID-19 epidemic to come to an end? | |
|  | A. Within one month |
|  | B. Within three months |
|  | C. Within six months |
|  | D. More than six months |
| Q16. How often are you worried about being infected by SARS-CoV-2? | |
|  | A. Seldom |
|  | B. Sometimes |
|  | C. Always |
| Q17. Do you think it necessary to timly release COVID-19 epidemic status? | |
|  | A. Very necessary |
|  | B. Necessary |
|  | C. I do not care |
|  | D. Not necessary |
| Q18. Which of the following do you think is the most disatrous for mankind? | |
|  | A. Traffic accident |
|  | B. Communicable diseases |
|  | C. War |
|  | D. Earthquake |
| Q19. What is your perceived likelihood of being infected with SARS-CoV-2? | |
|  | A. Very likely |
|  | B. Likely |
|  | C. Not very likely |
|  | D. Impossible at all |
| Q20. How much do you know about COIVD-19? | |
|  | A. Very much |
|  | B. A lot |
|  | C. A little |
|  | D. Nothing at all |
| Q21. What do you think is the psychological impact of COVID-19 on public? | |
|  | A. Panic |
|  | B. Scared |
|  | C. Nervous |
|  | D. Anxious |
| Q22. Where do you think is the most dangerous place during COVID-19 epidemic? | |
|  | A. Dental hospital |
|  | B. General hospital |
|  | C. Shopping mall |
|  | D. Market |
|  | E. Railway station |
|  | F. Airport |
| Q23. How much is your dental visit impacted by COVID-19 epidemic? | |
|  | A. Very much impacted |
|  | B. Impacted a lot |
|  | C. Somewhat impacted |
|  | D. Not impacted |
| Q24. What is your current degree of fearfulness toward dental visit? | |
|  | A. Very much |
|  | B. A little |
|  | C. Not at all |
| Q25. Do you know dental treatment generate droplets and aerosol? | |
|  | A. I know it very clearly |
|  | B. I know it clearly |
|  | C. I know a little about it |
|  | D. I know nothing about it |
| Q26. How much do you think unmaksing during dental treatment increases risk of infection? | |
|  | A. Serverly increased |
|  | B. Somewhat increased |
|  | C. No increase |
| Q27. Do you think oral health impacts general health? | |
|  | A. Yes, oral health problems could ultimately lead to death |
|  | B. Yes, oral health problems could lead to diseases in other parts of the body |
|  | C. No |
| Q28. Do you think taking medicines can cure your dental problems? | |
|  | A. Yes |
|  | B. Can partly alleviate symptoms |
|  | C. No |
| Q29. Which of the following helps you think positively towards COIVD-19 epidemic? | |
|  | A. Dedication by health care providers |
|  | B. Nation-wide joint effort to combat COVID-19 epidemic |
|  | C. Research and development of new therapies and vaccines |
|  | D. International assistance |
| Q30. Which of the following personal characteristics helps the most in enduring COVID-19 epidemic? | |
|  | A. Peaceful state of mind |
|  | B. Being able to adapt to environment |
|  | C. Being optimistic |
|  | D. Being cautious |
|  | E. Being self-disciplined |
|  | F. Being decisive |
|  | G. Trusting others |
| Q31. Which of the following relieves you the most? | |
|  | A. Epidemic weakens |
|  | B. My symptom gets better |
|  | C. My symptom is not severe |
|  | D. Support from friends and family |
| Q32. How much do you trust health care providers? | |
|  | A. Very much |
|  | B. Somewhat |
|  | C. No at all |
| Q33. Do you trust most people or exercise caution when getting along with others? | |
|  | A. Most people are trustworthy |
|  | B. Caution must be exercised |
| Q34. Do you think most people are helpful or only care about themselves? | |
|  | A. Most people are helpful |
|  | B. Most people only care about themseleves |
| Q35. Do you think others will take advantage of you or treat you fairly? | |
|  | A. Take advantage of me |
|  | B. Treat me fairly |
| When you confront setbacks, what is your attitude and coping method? | |
| Q36. Distract by working, studying, or some other activities | |
|  | A. Never |
|  | B. Occasionally |
|  | C. Sometimes |
|  | D. Always |
| Q37. Talk to others | |
|  | A. Never |
|  | B. Occasionally |
|  | C. Sometimes |
|  | D. Always |
| Q38. Try to see the positive aspect of everything | |
|  | A. Never |
|  | B. Occasionally |
|  | C. Sometimes |
|  | D. Always |
| Q39. Change your mindset and reconsider what is important in life | |
|  | A. Never |
|  | B. Occasionally |
|  | C. Sometimes |
|  | D. Always |
| Q40. Try not to take things too seriously | |
|  | A. Never |
|  | B. Occasionally |
|  | C. Sometimes |
|  | D. Always |
| Q41. Insist your own viewpoint and fight for what you want | |
|  | A. Never |
|  | B. Occasionally |
|  | C. Sometimes |
|  | D. Always |
| Q42. Try several different solutions | |
|  | A. Never |
|  | B. Occasionally |
|  | C. Sometimes |
|  | D. Always |
| Q43. Seek advice from relatives, colleagues, or classmates | |
|  | A. Never |
|  | B. Occasionally |
|  | C. Sometimes |
|  | D. Always |
| Q44. Change my perivous thoughts or some of my own problems | |
|  | A. Never |
|  | B. Occasionally |
|  | C. Sometimes |
|  | D. Always |
| Q45. Try to follow others' solutions | |
|  | A. Never |
|  | B. Occasionally |
|  | C. Sometimes |
|  | D. Always |
| Q46. Resort to my hobbies and actively participate in various activities | |
|  | A. Never |
|  | B. Occasionally |
|  | C. Sometimes |
|  | D. Always |
| Q47. Try to restrain disappointment, resentment, and anger | |
|  | A. Never |
|  | B. Occasionally |
|  | C. Sometimes |
|  | D. Always |
| Q48. Try to take a leave and forget about the worries for the moment | |
|  | A. Never |
|  | B. Occasionally |
|  | C. Sometimes |
|  | D. Always |
| Q49. To smoke, drink, taking drugs, and eating | |
|  | A. Never |
|  | B. Occasionally |
|  | C. Sometimes |
|  | D. Always |
| Q50. Time will change everything, the only thing to do is to wait | |
|  | A. Never |
|  | B. Occasionally |
|  | C. Sometimes |
|  | D. Always |
| Q51. Try to forget the whole thing | |
|  | A. Never |
|  | B. Occasionally |
|  | C. Sometimes |
|  | D. Always |
| Q52. To count on others to solve the issue | |
|  | A. Never |
|  | B. Occasionally |
|  | C. Sometimes |
|  | D. Always |
| Q53. Accept the reality because there is no other solution | |
|  | A. Never |
|  | B. Occasionally |
|  | C. Sometimes |
|  | D. Always |
| Q54. To dream of miracle | |
|  | A. Never |
|  | B. Occasionally |
|  | C. Sometimes |
|  | D. Always |
| Q55. To comfort yourself | |
|  | A. Never |
|  | B. Occasionally |
|  | C. Sometimes |
|  | D. Always |
| Q56. Are you normally an anxious person | |
|  | A. Yes |
|  | B. No |
| Q57. I spend a lot of time overcoming my shyness in new environment | |
|  | A. Very much like me |
|  | B. Somewhat like me |
|  | C. A little like me |
|  | D. Not at all like me |
| Q58. I appear awkward while working | |
|  | A. Very much like me |
|  | B. Somewhat like me |
|  | C. A little like me |
|  | D. Not at all like me |
| Q59. I am very likely to be embarrassed | |
|  | A. Very much like me |
|  | B. Somewhat like me |
|  | C. A little like me |
|  | D. Not at all like me |
| Q60. I am skilled at communicating with others | |
|  | A. Very much like me |
|  | B. Somewhat like me |
|  | C. A little like me |
|  | D. Not at all like me |
| Q61. I feel nervous talking to public | |
|  | A. Very much like me |
|  | B. Somewhat like me |
|  | C. A little like me |
|  | D. Not at all like me |
| Q62. A large population makes me nervous | |
|  | A. Very much like me |
|  | B. Somewhat like me |
|  | C. A little like me |
|  | D. Not at all like me |

**Supplementary Table 2.** Loadings of psychological factors on eigenvectors of responses among participants in group T-nV and C

|  |  | **PC1** | **PC2** | **PC3** | **PC4** | **PC5** | **PC6** | **PC7** | **PC8** | **PC9** |
| --- | --- | --- | --- | --- | --- | --- | --- | --- | --- | --- |
| **Domain A: Perception towards COVID-19** | | | | | | | | | | |
|  | Q16: Worried about being infected | -0.391 | -0.018 | **-0.492** | -0.186 | 0.319 | **0.432** | -0.026 | 0.517 | -0.111 |
|  | Q19: Likelihood of being infected | **0.453** | -0.031 | -0.061 | 0.259 | -0.077 | -0.025 | 0.696 | 0.481 | 0.006 |
| **Domain B: Perception towards dental attendance during COVID-19 epidemic** | | | | | | | | | | |
|  | Q23: Impact on dental attendance | **0.430** | 0.316 | 0.050 | 0.123 | 0.047 | 0.113 | -0.563 | 0.378 | 0.473 |
|  | Q24: Fearfulness towards dental visit | 0.235 | 0.107 | 0.335 | -0.764 | 0.436 | -0.132 | 0.158 | 0.073 | -0.004 |
|  | Q26: Unmasking during dental treatment | **0.420** | 0.370 | -0.077 | 0.068 | 0.070 | **0.478** | -0.057 | -0.292 | -0.595 |
|  | Q27: Oral health impacts general health | 0.114 | 0.356 | **-0.668** | -0.360 | -0.333 | -0.204 | 0.131 | -0.232 | 0.246 |
| **Domain C: Trust** | | | | | | | | | | |
|  | Q35: Trust towards health care providers | -0.231 | 0.468 | -0.002 | 0.367 | 0.585 | -0.046 | 0.283 | -0.271 | 0.307 |
| **Domain D: Coping** | | | | | | | | | | |
|  | Positive coping inventory | 0.268 | -0.549 | -0.135 | -0.059 | 0.167 | **0.484** | 0.101 | -0.373 | 0.440 |
| **Domain E: Others** | | | | | | | | | | |
|  | Social anxiety inventory | -0.295 | 0.320 | **0.410** | -0.167 | -0.462 | **0.526** | 0.249 | 0.030 | 0.248 |

All questions with significant differences between group T-nV and group C, except for those with categorical responses, were included for PCA. Questions with loadings not less than 0.4 were bolded in PCs significantly associated with unnecessary dental avoidance.

**Supplementary Table 3.** Loadings of psychological factors on eigenvectors of responses among participants in group nT-V and C

|  |  | **PC1** | **PC2** | **PC3** | **PC4** | **PC5** | **PC6** |
| --- | --- | --- | --- | --- | --- | --- | --- |
| **Domain A: Perception towards COVID-19** | |  |  |  |  |  |  |
|  | Q15: Expected duration of the epidemic | **-0.412** | 0.379 | -0.130 | 0.568 | 0.516 | -0.286 |
|  | Q16: Worried about being infected | **-0.566** | -0.033 | 0.043 | -0.169 | 0.202 | 0.779 |
|  | Q19: Likelihood of being infected | **0.467** | -0.342 | -0.145 | -0.130 | 0.786 | 0.101 |
| **Domain B: Perception towards dental attendance during COVID-19 epidemic** | | | | | | | |
|  | Q23: Impact on dental attendance | **0.442** | 0.089 | 0.273 | 0.683 | -0.124 | 0.490 |
|  | Q24: Fearfulness towards dental visit | 0.184 | **0.662** | 0.575 | -0.385 | 0.223 | -0.012 |
| **Domain C: Trust** | | | | | | | |
|  | Q35: Trust towards health care providers | -0.252 | **-0.541** | 0.745 | 0.131 | 0.104 | -0.245 |

All questions with significant differences between group T-nV and group C, except for those with categorical responses, were included for PCA. Questions with loadings not less than 0.4 were bolded in PCs significantly associated with unnecessary dental attendance.
